# Micron-sized iron oxide particles for both MRI cell tracking and magnetic fluid hyperthermia treatment

**DOI:** 10.1038/s41598-021-82095-6

**Published:** 2021-02-08

**Authors:** Laurence Dallet, Dimitri Stanicki, Pierre Voisin, Sylvain Miraux, Emeline J. Ribot

**Affiliations:** 1 Centre de Résonance Magnétique des Systèmes Biologiques, UMR 5536, CNRS/Univ. Bordeaux, 146 rue Léo Saignat, 33076 Bordeaux, France; 2 Department of General, Organic and Biomedical Chemistry, NMR and Molecular Imaging Laboratory, University of Mons, 19 avenue Maistriau, 7000 Mons, Belgium

**Keywords:** Cancer therapy, Biophysics, Endocytosis, Magnetic resonance imaging, Imaging

## Abstract

Iron oxide particles (IOP) are commonly used for Cellular Magnetic Resonance Imaging (MRI) and in combination with several treatments, like Magnetic Fluid Hyperthermia (MFH), due to the rise in temperature they provoke under an Alternating Magnetic Field (AMF). Micrometric IOP have a high sensitivity of detection. Nevertheless, little is known about their internalization processes or their potential heat power. Two micrometric commercial IOP (from Bangs Laboratories and Chemicell) were characterized by Transmission Electron Microscopy (TEM) and their endocytic pathways into glioma cells were analyzed. Their Specific Absorption Rate (SAR) and cytotoxicity were evaluated using a commercial AMF inductor. T2-weighted imaging was used to monitor tumor growth in vivo after MFH treatment in mice. The two micron-sized IOP had similar structures and r_2_ relaxivities (100 mM^−1^ s^−1^) but involved different endocytic pathways. Only ScreenMAG particles generated a significant rise in temperature following AMF (SAR = 113 W g^−1^ Fe). After 1 h of AMF exposure, 60% of ScreenMAG-labeled cells died. Translated to a glioma model, 89% of mice responded to the treatment with smaller tumor volume 42 days post-implantation. Micrometric particles were investigated from their characterization to their intracellular internalization pathways and applied in one in vivo cancer treatment, i.e. MFH.

## Introduction

Magnetic Resonance Imaging (MRI) has become the reference imaging technique for the diagnosis and prognosis of many cancers. It is also an ideal biomedical imaging technique for monitoring therapeutic efficiencies. In addition, cellular MRI enables the detection and tracking of cells within the whole body. Due to the low sensitivity of this imaging technique, iron oxide particles (IOP) are usually employed to label the cells of interest. The efficacy of IOP as contrast agents can be evaluated through the ratio *r*_2_/*r*_1_*,* where *r*_1_ and *r*_2_ represent the longitudinal and transverse relaxivities, respectively. For T2 contrast agents, a high ratio indicates a better contrast efficiency. Several micron-sized particles (diameter > 750 nm) were synthesized in order to increase this ratio and consequently increase the sensitivity of detection. The relaxivities of some nanoparticles and microparticles at high field are summarized in Table 1. The *r*_2_ is relatively the same for nanoparticles and microparticles, however, micrometric-sized particles have key advantages over nanoparticles. Their larger surface enables more moieties (like antibodies, aptamers, etc.) to be grafted per particle^1^ in order to increase targeting efficiency^2^. Also, due their large size and the high iron content per particle, single particles can be detected by T2*-weighted MRI^3^, which is essential for cell tracking over long periods of time. In parallel, single cancer cells labeled with micrometric particles have been detected in mice^3–5^.

**Table 1:** Relaxivities of various particles at high magnetic fields.

IOP	Diameter (nm)	Field	r_1_ (mM^−1^ s^−1^)	r_2_ (mM^−1^ s^−1^)	r_2_/r_1_	References
PLGA microparticles	1500–2100	4 T		93–20.9		Nkansah et al.^24^
MEDG002 (Bangs Laboratories)	1600	4 T		52.7		Nkansah et al.^24^
Ferumoxtran-10 (Guerbet)	30	4.7 T	5.08	82.2	16.2	Antell H et al.^60^
Ferumoxytol (AMAG Pharmaceuticals)	24	3 T	10.0	62.3	6.2	Knobloch et al.^61^
		7 T	2.12	33.15	15.6	Gharagouzloo et al.^62^
Endorem (Guerbet)	150	4. 7 T	2.11	113.99	54	Ali LMA et al.^63^
Feridex (Advance Magnetics)		4 T		110.5		Nkansah et al.^24^
IOP coated with aminosilane (Chemicell)	50	3 T	6.1	53	8.7	Mamani et al.^64^
P904 (Guerbet)	30	4.7 T	4	92	23	Guerbet

The names, sizes and relaxivities are shown at the corresponding magnetic fields.

Micron-sized particles can be designed and synthesized by expert chemistry labs^6–8^ in order to utilize them for a specific application or for targeting specific cells. Also, specialist companies have available several large commercial micrometric IOP that are accessible. One of their advantages is supposed to be the reproducible synthesis leading to large batches. In vitro and in vivo^9^ studies have shown the possibility of using micrometric IOP from Chemicell for mesenchymal stem cell monitoring by MRI. Also, micrometric IOP from Bangs Laboratories have been used for cancer cell tracking^4,10^. These commercial IOP have interesting properties, including an efficient internalization, biocompatibility, long stability within cells and, of course, a high sensitivity of detection by MRI.

Nevertheless, to our knowledge, no studies have been performed to assess the internalization pathways of these commercial micron-sized particles within cancer cells. The precise knowledge of the endocytosis mechanism is important for the biomedical application and the labeling of cells of interest.

Another application of IOP in the biomedical field is their use for treatment. IOP have shown to potentiate the efficiency of multiple therapies like cryoablation^11^ and radiotherapy due to their radiosensitizing properties^12^. IOP limit healthy neighboring tissue damage during High Intensity Focused Ultrasound (HIFU) experiments and shorten Focused Ultra-Sound application^13,14^. Also, IOP have been used for Magnetic Fluid Hyperthermia (MFH) in combination with alternating magnetic fields (AMF). This principle is based on the ability of IOP to heat under AMF exposure due to thermal losses of the dynamic magnetization loops. Once the IOP are co-localized with tumor cells, a rise in temperature following AMF application is generated, leading to cytotoxicity. It has been shown that the increase in sizes and concentrations of the IOP has a positive effect on temperature distribution in the tissue^15^. The efficiency of MFH therapy is dependent on the Specific Absorption Rate (SAR) of the IOP employed. The SAR of nanoparticles dedicated to MFH application can reach a few hundred W/g(Fe)^16,22^, although the SAR of Ferumoxytol (60 W/g(Fe)^17^), which is the only IOP that can be used off-label as an MRI contrast agent, is very low^18^. No study has previously evaluated if the commercial micrometric particles generate a high SAR. We hypothesized that due to the high amount of iron per particle, large IOP can also be beneficial for MFH treatment.

Consequently, the goal of our study was to investigate if micrometric particles would be good candidates for both MRI cell tracking and as a one-treatment strategy against glioma, i.e. MFH in vivo. Particles were characterized by Transmission Electron Microscopy (TEM) and MRI. Their specific endocytic pathways involved in the internalization into a human glioma cell line were determined. Then, in vitro experiments were performed in the presence of an AMF. Finally, the MFH treatment was applied on a mouse model of human glioma under MRI control.

## Results

### Characterization of the iron oxide particles

**Figure 1:**
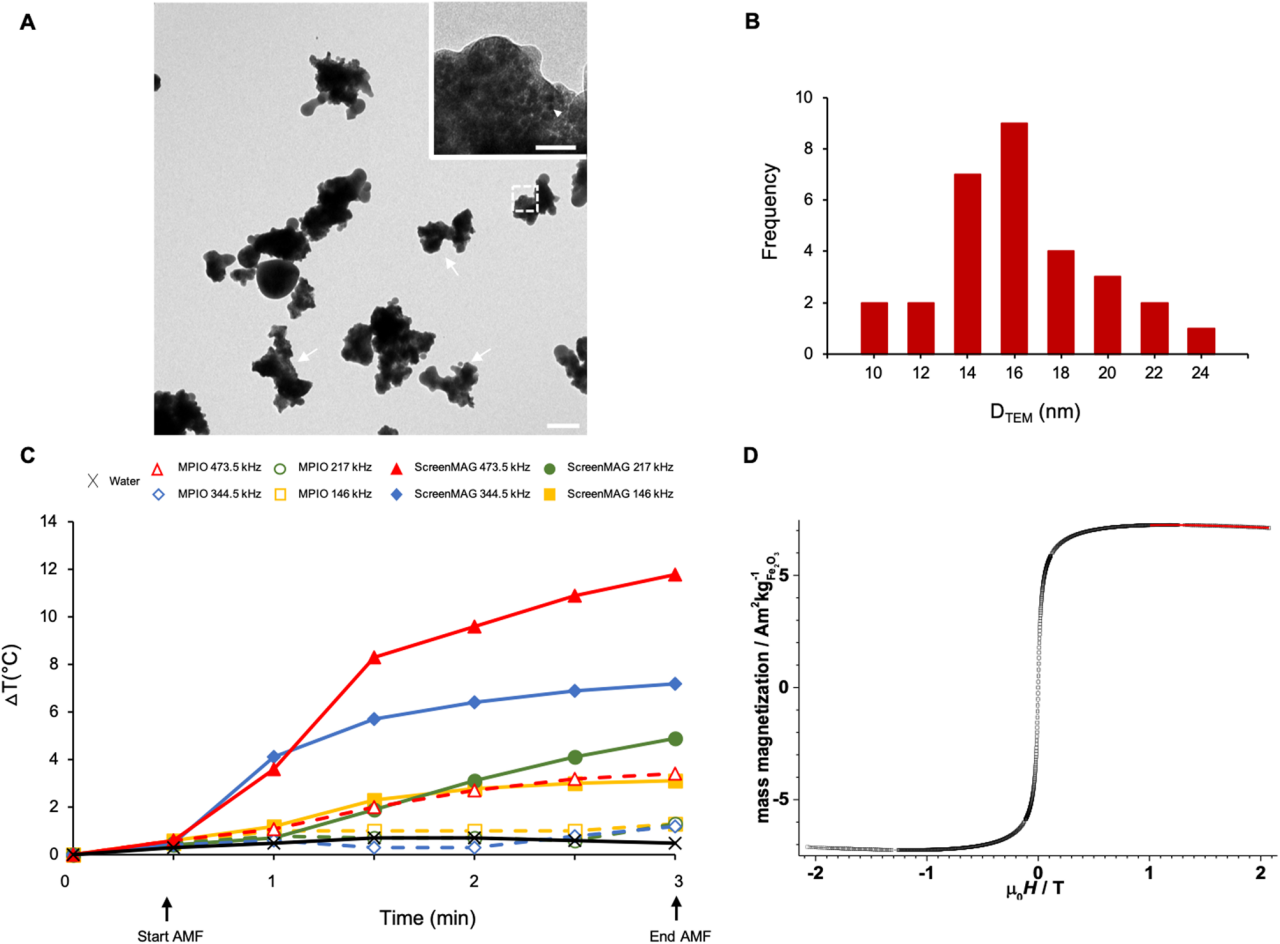
Characterization of the commercial micrometric iron oxide particles ScreenMAG (carboxyl-functionalized magnetic fluorescent silica particles; λ_exc_ = 502 nm; λ_em_ = 525 nm). (**A**) Transmission electron microscopy (TEM) images of ScreenMAG. Sizes and shapes were observed in negative staining. White arrows indicate one iron oxide particle. Particles are assembled as multi-iron oxide cores inside a matrix. White head arrows show single iron oxide cores. Scale bar: 500 nm (insert: 100 nm). (**B**) Histogram of the iron core diameters determined by TEM. (**C**) Temperature elevation curves of aqueous suspensions of MPIO or ScreenMAG at 57 mM Fe after application of an alternating magnetic field (AMF) at different combinations of frequencies and amplitudes: 146 kHz, 21.90 kA m^−1^; 217 kHz, 20.69 kA m^−1^; 344.5 kHz, 16.23 kA m^−1^ and 473.5 kHz, 13.36 kA m^−1^. (**D**) Field dependence of the mass magnetization of ScreenMAG at 296 K (black squares). The law of approach fit is reported as a red line. The electromagnet soft iron poles separation was reduced to 5 mm to allow maximum field (2.1 T). Samples were weighed accurately with a Mettler MX5 microbalance. 30 µL of the ScreenMAG stock solution was sealed in a light tin capsule for liquid samples for elemental analysis. An empty capsule was measured beforehand and the resulting diamagnetic contribution convoluted with the quartz rod was accordingly subtracted from the measurements. Magnetization values were normalized respectively to the Fe cation content (265 mM) as determined by ICP analysis: 30 µL corresponding thus to 0.444 mg of Fe and 0.635 mg of maghemite.

Physicochemical characteristics of the IOP were determined using negative staining TEM. The sizes, shapes and structures of each particle sample were identified (Fig. 1A and Supplementary Data S1A). At low magnification, ScreenMAG and MPIO particles are of irregular shapes. MPIO particles (Supplementary Data S1A) are heterogeneous with a range of diameters from 50 nm to 1.65 µm, whereas the sizes of the ScreenMAG particles (Fig. 1A) are more homogeneous with a mean diameter of 900 nm. The size of each IOP sample is in accordance with the commercial specification data sheets. For both particle types, higher magnification images showed that multiple small iron oxide nanodomains (white head arrows in Fig. 1A) were clustered in the different matrices. The mean sizes of the magnetic cores of ScreenMAG were measured at 15.8 ± 0.6 nm (Fig. 1B).

The iron concentrations of these particles were determined by Inductively Coupled Plasma-Atomic Emission Spectroscopy (ICP-AES). Concentrations of 56.8 ± 1.4 mM and 265.5 ± 7.7 mM were found for the commercial stock solutions of MPIO and ScreenMAG particles, respectively.

Longitudinal and transverse relaxivities (r_1_ and r_2_) for MPIO and ScreenMAG were measured at 4.7 T: r_1_ = 11.36 mM^−1^ s^−1^ and 13.46 mM^−1^ s^−1^; r_2_ = 111.0 mM^−1^ s^−1^ and 102.3 mM^−1^ s^−1^, respectively. The *r*_2_/*r*_1_ was 7.6 and 9.8 for ScreenMAG and MPIO, respectively.

Figure 1C shows that the macroscopic heating efficiency was dependent on the frequency: the higher the frequency, the higher the SAR value. Among the multiple magnetic field frequencies tested, the combination 473.5 kHz and 13.4 kA m^−1^ of amplitude generated the largest macroscopic heating. At the same concentration, compared to MPIO that generated a less than 4 °C rise of temperature, the ScreenMAG particles generated a substantial heat increase of 12 °C in 3 min. The elevation of temperature reached a plateau after 9 min of applying AMF (Supplementary Data S2A).

Based on these results, a SAR of 113 W g^−1^ Fe ± 13 W g^−1^ Fe was measured in water for the ScreenMAG particles under an AMF of 473.5 kHz frequency and 13.4 kA m^−1^ amplitude, compared to 37 W g^−1^ Fe ± 3 W g^−1^ Fe for MPIO particles.

SAR measurements were also performed in media with different viscosities (Supplementary Data S2B). ScreenMAG SAR decreased from 113 to 70 W g^−1^ Fe when the media viscosity increased.

Hysteretic measurement of the ScreenMAG particle suspension evidences superparamagnetic behavior at room temperature (Fig. 1D), with saturation effective above 0.5 T. The fit, reported in red in Fig. 1D, gave a saturation value *M*_*S*_ of 7.69 ± 0.02 ${\text{Am}}^{2}{\text{kg}}_{{\text{Fe}}_{2}{\text{O}}_{3}}^{-1}$ (0.1178 ± 0.0002 μ_B_/Fe). This magnetization is significantly lower than the values found in the bulk (60–80 ${\text{Am}}^{2}{\text{kg}}_{{\text{Fe}}_{2}{\text{O}}_{3}}^{-1}$ corresponding to 0.86–1.14 μ_B_/Fe), pointing towards a rather small average core size. This is in agreement with the core diameters previously measured on the TEM images.

The ScreenMAG colloidal dispersion was measured using Dynamic Light Scattering (DLS), and proved to be stable for 1 h with a mean hydrodynamic diameter averaging during that period of 774 ± 151 nm (Supplementary Data S3B), which is in agreement with the global size of the particles measured in TEM. The measured Z-average diameter started to decrease approximately linearly over time, with a rate of about 0.2 nm s^−1^. Concomitantly, the mean backscattered light intensity was constant for 1 h (mean: 2157 ± 94 kcounts s^−1^) and then started to decrease. A decay time was estimated graphically at 10,800 s, meaning the characteristic sedimentation time of the dispersion is approximately 3 h.

### Cell internalization of the iron oxide particles

**Figure 2:**
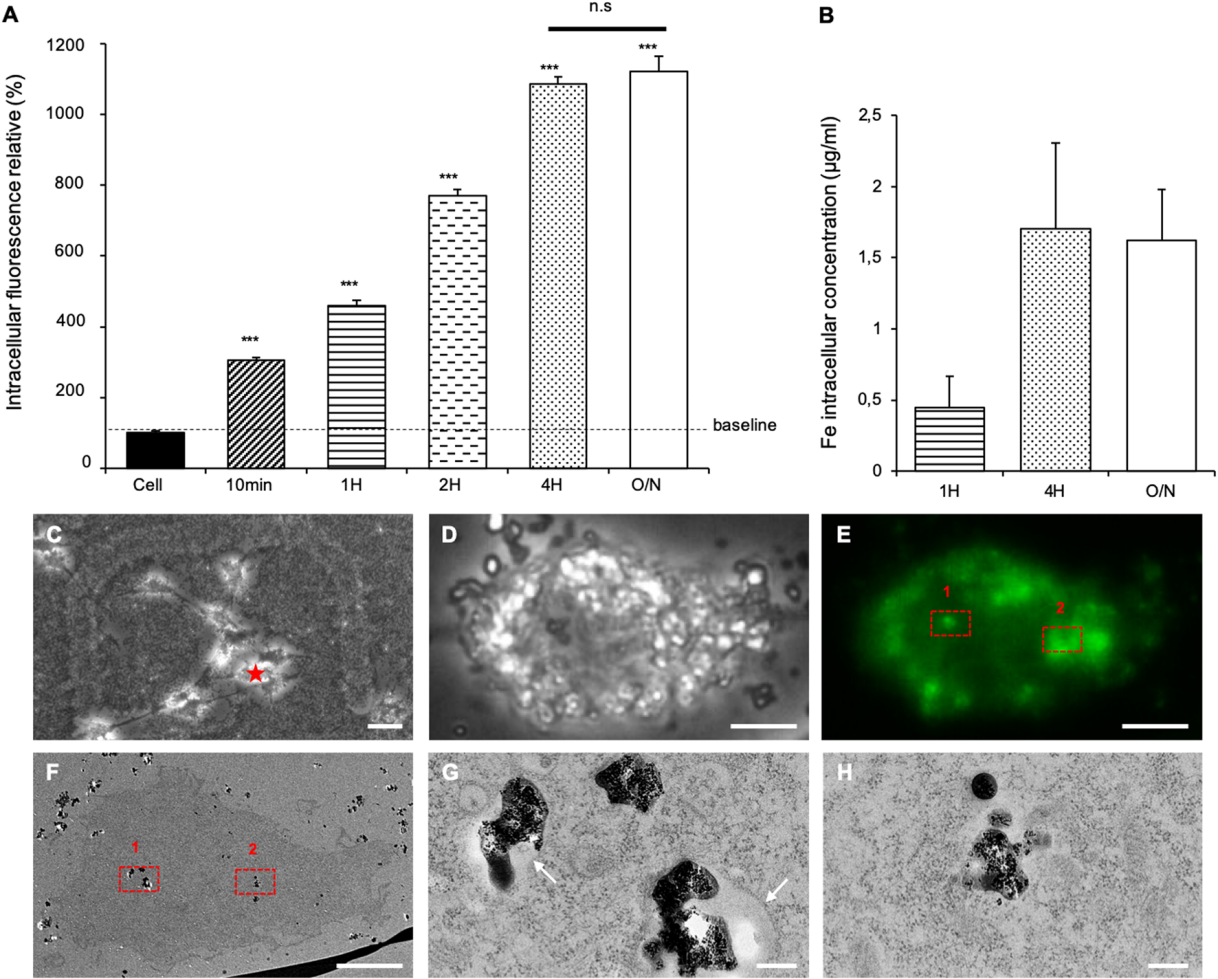
Analysis of the intracellular delivery of ScreenMAG particles in U87-MG cells. (**A**) Kinetic of ScreenMAG internalization quantified by flow cytometry (λ_exc_ = 502 nm; λ_em_ = 525 nm). Iron oxide particles (IOP) were incubated during different periods (10 min, 1, 2, 4 h or overnight (O/N)) and intracellular fluorescent relatives were quantified. ***Significantly different from the control (*P* ≤ 0.01). (**B**) Fe intracellular concentration determined by an iron assay kit (Abnova, KA0814) at 593 nm. IOP were incubated during different periods (1 h, 4 h or overnight) before the iron concentrations were determined. (**C**–**F**) Correlative Light Electron Microscopy (CLEM) was used. Both fluorescence and electron microscopies were performed on the exact same cell (star in **C**). (**C**) Lower magnification of cells in light microscopy. (**D**) Phase contrast and (**E**) Fluorescent image of a ScreenMAG-labeled U87-MG cell. (**F**) TEM section of the same cell. (**G**,**H**) Enlargements correspond to the two regions of interest (ROI) [(red squares 1 and 2 respectively in (**E**) and (**F**)]. White arrows indicate the vesicular membranes. Scale bars are 40 μm (**C**), 5 μm (**D**,**E**,**F**) and 200 nm (**G**,**H**).

A kinetic study of the intracellular fluorescence (Fig. 2A) demonstrated that a significant increase of the intracellular fluorescence was observed only 10 min after the incubation of the ScreenMAG with the cells, suggesting that these particles were internalized very rapidly. Intracellular fluorescence increased when the incubation period was maintained for 4 h, and remained similar after an overnight incubation. This uptake was confirmed using a correlative microscopy approach (Fig. 2C–H). In fluorescent microscopy, strong “punctuated” fluorescent signals were observed inside the ScreenMAG-labeled cells. In TEM, dense-electron structures were identified and correlated with the intense fluorescent signal (Fig. 2E,F). An example is shown in the red squares 1 and 2 (Fig. 2G,H). The IOP aggregates were found surrounded by endocytic membrane (Fig. 2G), which localized in perinuclear areas but never inside the cell nucleus (Fig. 2E). Also, the amount of internalized iron increased when the incubation time lengthened (Fig. 2B), which is in agreement with the intracellular fluorescence study. After 4-h and overnight incubations, similar amounts of 1.6 ± 0.4 μg ml^−1^ of intracellular Fe were measured. In addition, HSP70 protein levels were assessed by western blot (Supplementary Data S4A–D) to determine if this internalization could induce a cellular stress. Similar HSP70 protein levels were measured in unlabeled cells and over the different IOP incubation conditions (*P* = 0.974). Furthermore, the proliferation rates of unlabeled and ScreenMAG-labeled cells were measured, and were found to be the same (Supplementary Data S5B).

**Figure 3:**
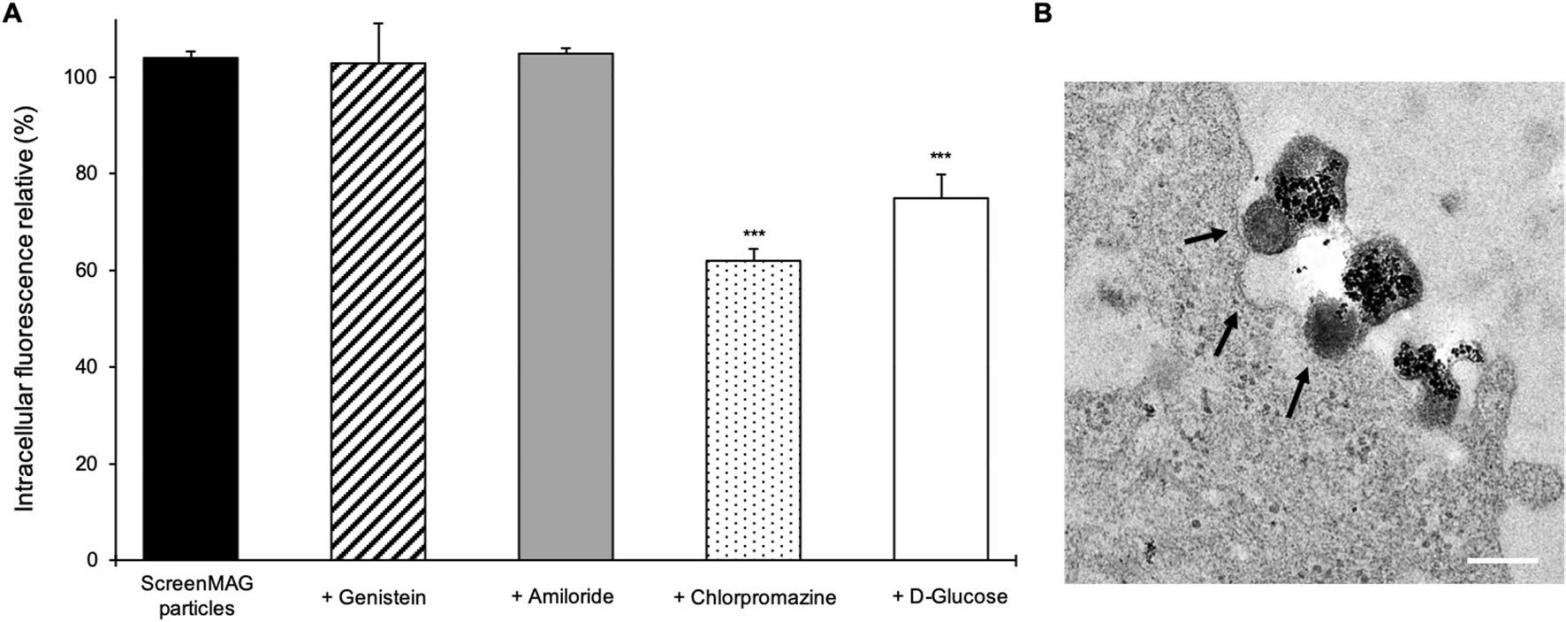
Internalization pathway of ScreenMAG particles in U87-MG cells. (**A**) Intracellular fluorescence analysis of iron oxide particles internalization within U87-MG cells treated with various chemical inhibitors (300 μM, 10 nM, 20 μM and 5 μM for genistein, amiloride, chlorpromazine and d-glucose, respectively) preventing different pathways of endocytosis. ***Significantly different from control (*P* ≤ 0.01). (**B**) Transmission electron microscopy image of a cell after 4 h of incubation with the iron oxide particles. Vesicles resembling to clathrin coating pits (dark arrows) are observed. Scale bar: 200 nm.

To explore the endocytic pathway, chemical inhibitors such as genistein, amiloride, chlorpromazine and 2-Deoxy-d-Glucose were used due to their well-known preferential perturbations of caveolae-mediated endocytosis, macropinocytosis, clathrin-mediated endocytosis and metabolic pathways, respectively. Inhibitors were used at suitable concentrations as determined by a cell viability assay (Supplementary Data S5C). These concentrations were used to specifically inhibit endocytic markers of each pathway. Intracellular ScreenMAG particles were significantly reduced by chlorpromazine treatment (*P* < 0.01). An inhibition of 38% was noted, while genistein and amiloride treatments had no effect (Fig. 3A). Furthermore, a decrease of 25% was observed with the metabolic inhibitor treatment. Consequently, the cellular uptake of ScreenMAG mainly involved clathrin-dependent endocytosis. In order to correlate the involvement of this internalization pathway, cells labeled with these particles were analyzed by TEM (Fig. 3B). The particles were detected near or inside vesicles created by plasma membrane. These vesicles presented electron-dense regions at their surface. The shapes and sizes of these structures resembled clathrin-coated pits, confirming the endocytic pathway.

The same experiments were performed with the MPIO particles. Macropinocytosis and caveolae-dependent pathways are two independent processes found to be involved in the internalization of these particles: inhibition of 25% and 37% were measured with amiloride and genistein, respectively. Furthermore, when amiloride and genistein treatment were incubated together, a fluorescence decrease of 59% was observed in U87-MG cells. The metabolic inhibitor treatment induced an inhibition of 69% (Supplementary Data S1B). In TEM images, MPIO particles were found in very large and small vesicles resembling macropinosomes and caveolae (Supplementary Data S1C).

### In vitro magnetic fluid hyperthermia experiments

**Figure 4:**
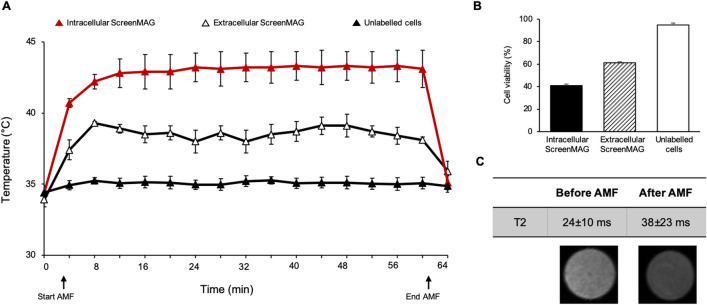
In vitro AMF application and corresponding MRI. (**A**) Temperature curves after application of an alternating magnetic field (AMF) (473 kHz and field strength of 13 kA m^−1^) (black arrow) on ScreenMAG-labeled U87-MG cells, extracellular ScreenMAG and unlabeled cells. (**B**) Cell viability of intracellular ScreenMAG, extracellular ScreenMAG and unlabeled cells after AMF application during 1 h. (**C**) T2 images and values before and after AMF of ScreenMAG-labeled cells (2 × 10^6^ cells) at 4.7 T. T2 values of unlabeled cells is 134 ms ± 6 ms.

ScreenMAG-labeled U87-MG cells, U87-MG unlabeled cells with ScreenMAG particles located in the extracellular medium or unlabeled cells alone were exposed to an AMF at 473.5 kHz frequency and 13.4 kA m^−1^ amplitude for 1 h (Fig. 4A). The temperature measured within the suspension containing ScreenMAG-labeled cells increased to 43.1 °C ± 0.1 °C, whereas extracellular ScreenMAG only moderately increased the temperature of the suspension to 38.6 °C ± 0.1 °C.

After 1 h of AMF exposure, a cell mortality of 59% ± 1% was observed when ScreenMAG were internalized compared to 39% ± 1% when the ScreenMAG were extracellular. No rise in temperature was observed in the unlabeled cells samples, leading to only 5% ± 2% of cell death (Fig. 4B).

In addition, the transverse relaxation time T2 before and after AMF application was measured at 4.7 T (Fig. 4C). Before AMF, the T2 value of 200,000 ScreenMAG-labeled cells was 24 ms ± 10 ms; after AMF, the T2 of the samples significantly lengthened to 38 ms ± 23 ms (*P* < 0.05). We estimated the amount of particles per cell based on the previously measured r_2_ relaxivity of ScreenMAG (102.3 mM^−1^ s^−1^). The T2 value of 24 ms obtained when cells were not lyzed can be reached by an iron concentration of 0.407 mM ± 0.18 mM ((1/102.3) × (1/0.024)). Knowing that the volume of 200,000 cells corresponds to 13 μL (average volume of one cell 65,000 μm^3^, measured on 20 cells by phase microscopy), and the molar mass of iron (159.69 g mol^−1^), 0.85 μg of iron in total can be deducted (0.407 × 10^–3^ × 159.69 × 13 × 10^–6^). Thus, for 1 cell, this led to 4.3 pg ± 1.9 pg of iron. According to the manufacturer, each particle encapsulates approximately 0.46 pg of iron; therefore, the average cell loading was estimated at 9 ± 4 ScreenMAG particles per cell.

### In vivo MFH treatment and MRI follow-up

**Figure 5:**
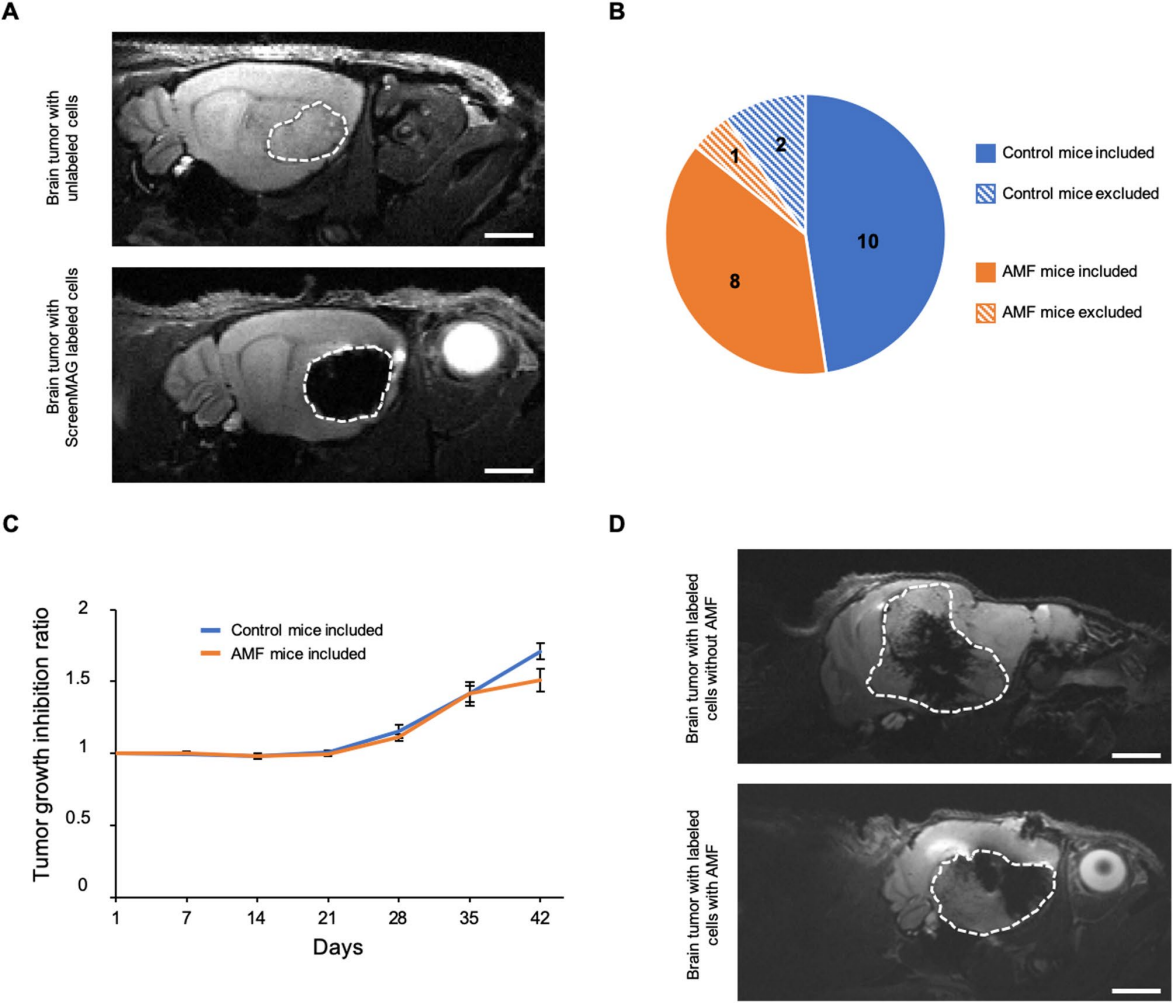
In vivo Magnetic fluid hyperthermia treatment. (**A**) T2-weighted MR images of mouse brains bearing intracranial tumor 7 days after the injection of unlabeled (top) or ScreenMAG-labeled (bottom) cells. White areas delimit the tumors. Scale bar: 6.5 mm. The tumor was delineated due to (1) its hypo-intense signal and (2) its hyper-intense signal compared to the surrounding healthy brain on T2-weighted images. Iron-labeled cells generate a hypo-intense signal on T2-weighted images^65^. On the other side, unlabeled tumors are depicted as hyper-intense areas in the brain on T2-weighted images^38^. (**B**) The pie diagram represents the numbers of mice included and excluded in the statistical analysis. (**C**) Tumor volume inhibition ratio of the Alternating Magnetic Field (AMF)-treated group (orange) and control group (blue) over 42 days. (**D**) T2-weighted MR images of mouse brains bearing ScreenMAG-labeled intracranial tumors, 42 days after AMF application (bottom) or not (top). White areas delimit the tumors. Scale bar: 6.5 mm.

Figure 5A shows a T2-weighted MR image of a mouse with an intracranial tumor after the injection of unlabeled (top) or ScreenMAG-labeled (bottom) U87-MG cells. A dense dark area revealed the presence of ScreenMAG-labeled glioma cells at day 7.

Mice with ScreenMAG-labeled U87-MG cells were then subject to MFH (AMF group) or not (control group). Based on our in vitro results and the necessity to maintain a high temperature within cells for at least 30 min^19^, mice received AMF for 1 h at 473.5 kHz frequency and 13.4 kA m^−1^ amplitude. During MFH, the rectal temperature of the mice did not increase more than 38 °C. MRI was used to measure the tumor volume once a week. Figure 5B represents the number of mice included or excluded from the statistical analysis. Based on the exclusion criteria, only 2 mice in the control group and 1 mouse in the AMF group were excluded due to abnormal tumor growth inhibition ratio (T/C). Figure 5C depicts T/C according to the two mice groups. At 42 days, a weak diminution of tumor growth was observed in the AMF group, even though no statistical significance was calculated (*P* = 0.078). A representative example of MR images of the tumors of each group at 42 days can be seen in Fig. 5D. A smaller tumor volume in mice receiving the treatment could be measured compared to control mice.

**Figure 6:**
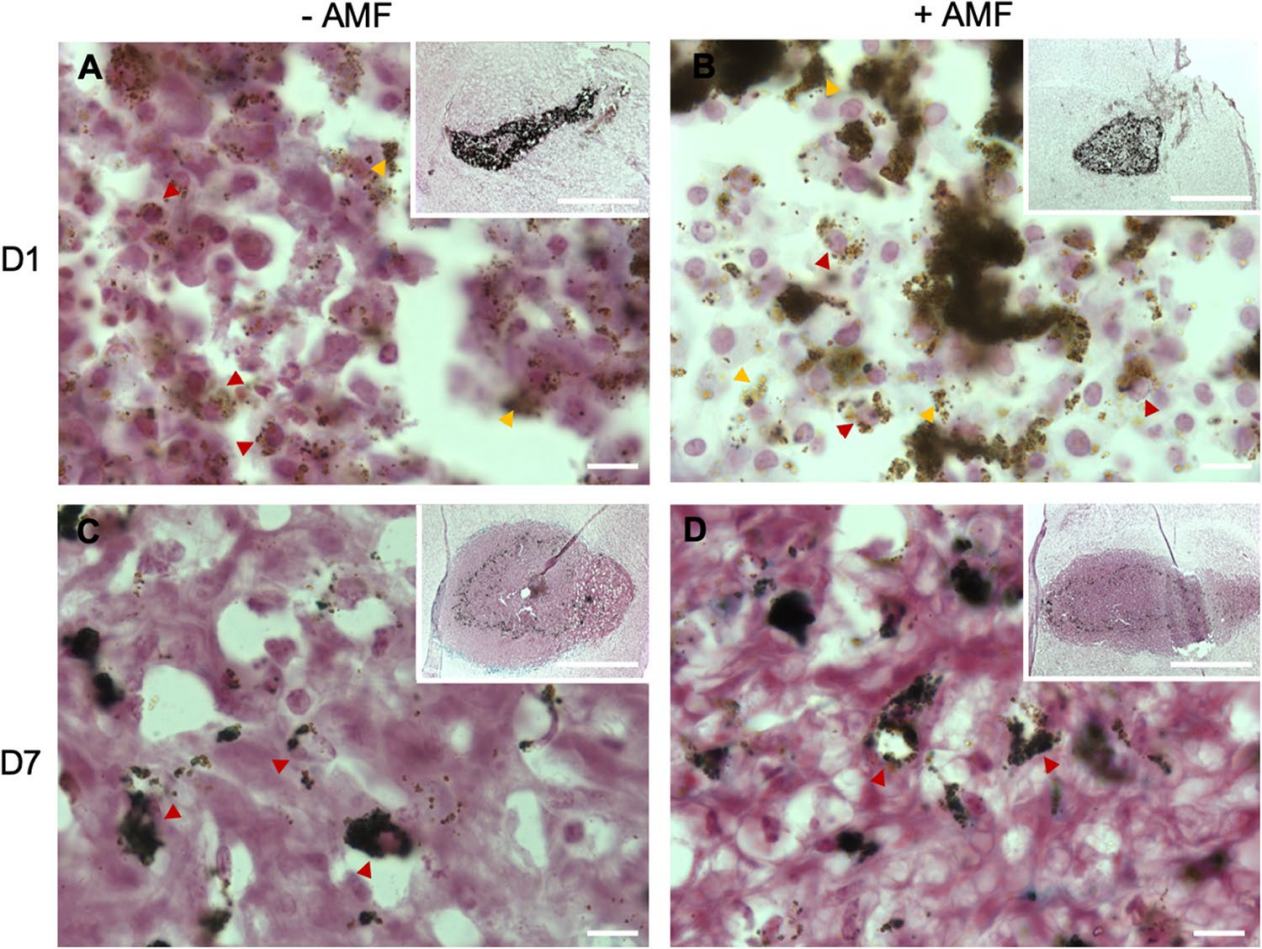
Images of iron staining on brain tumor sections. The staining was performed on brain tumors extracted 1 day (**A**,**B**) or 7 days (**C**,**D**) after Alternating Magnetic Field exposure (**B**,**D**) or not (**A**,**C**). Red arrows indicate intracellular ScreenMAG particles. Yellow arrows indicate extracellular ScreenMAG particles. Scale bar: 20 μm (insert: 1 mm).

Qualitative analysis of intracellular iron was performed on brain slides of mice submitted or not to AMF application (Fig. 6A–D). Although iron was mostly detected within cancer cells when no AMF was applied, the ScreenMAG particles were found equally distributed in the extracellular and intracellular spaces one day after AMF application. However, 7 days after the AMF application, IOP were mainly detected within the cells.

## Discussion

This present research studied the complete process of micrometric particles—from particle characterization to intracellular internalization pathway and in vivo application—to evaluate if a micrometric-sized particle is valuable for MFH treatments.

The TEM images could be used to fully characterize two commercial IOP. MPIO and ScreenMAG are clusters of IOP within a polymer matrix, exhibiting superparamagnetic states. This information is really important for applications in magnetic hyperthermia. Indeed, recent studies have shown that iron oxide multi-core particles are more efficient for magnetic hyperthermia^20–22^ compared to mono-core ones. These magnetic multi-domains facilitate the retention of superparamagnetic behavior. Such multi-domain particles are very useful because transverse relaxivities for MRI contrast generation are enhanced^23^. Indeed, an r_2_ value close to 100 mM^−1^ s^−1^ was measured at 4.7 T, which is in agreement with other microparticles^24^ (Table 1). Even though ScreenMAG and MPIO particles are good T2 and T2* contrast agents, they have potential to also be used as T1 contrast agents. This may be an advantage in measuring their MFH-necessary high concentrations in vivo^25^ compared to T2 or T2* imaging. In addition, it can clarify their depiction as they would not be falsely attributed to other T2* generating species such as air or hemorrhage.

The internalization of these two particles into glioma cells involves, in majority, energy-dependent mechanisms. It was well established that routes of internalization are size-dependent^26^. The internalization pathways of MPIO particles are in agreement with the heterogeneous particle sizes observed in negative staining. Our results suggest that the MPIO with the smallest diameters (~ 50 nm) are internalized by caveolae-dependent pathways, whereas the larger MPIO particles (~ 1 µm) are internalized by macropinocytosis. These classic internalization processes are present in all cell types and could be involved in other cancer cells. Interestingly, even though the size of ScreenMAG is relatively large (1 µm), our results indicate that the internalization of these particles mainly involves the clathrin-dependent endocytic pathway. The size of the particles passing through the clathrin-dependent pathway is still controversial. A recent study showed that it depends on the cell type. The authors demonstrated that the clathrin-dependent endocytic pathway leads to a more efficient uptake into cancer cell lines than non-tumor cell lines^27^. This may be due to the fact that cancer cell lines usually express higher amounts of clathrins.

Our DLS measurement showed that the ScreenMAG particles are relatively stable over 1 h. The particles may stay in suspension in the culture media temporarily and then probably deposit on the bottom of the well and decrease in diameter size during the incubation time. This instability may be due to the presence of salt in the media, which could modify the colloidal stability of the IOP^28^. This may explain the importance of the clathrin pathway in the internalization of the ScreenMAG particles, compared to the expected macropinocytosis pathway. A more detailed study is necessary to determine the exact location of the ScreenMAG particles in the glioma cells to confirm this hypothesis. Nevertheless, the involvement of the clathrin pathway is interesting as it can provide a selective internalization route via a receptor-mediated uptake. This may be used for targeting drug-loaded IOP^29^. Indeed, Gao et al.^30^ designed nanoparticles to be selectively endocytosed into glioma cells through the clathrin pathways. Surface modifications of the micron-sized particles tested here could also lead to this labeling selectivity. However, surface modifications could also modify the colloidal stability^31^, and the endocytic mechanism.

The presence of the IOP under clusters, as in the case with ScreenMAG particles, is very high. Several studies have already shown that this formation of clusters leads to very good MFH treatment efficiencies^32–34^. Their internalization did not affect cell proliferation nor induce cellular stress. The cellular uptake estimated from MRI relaxivities is comparable to other studies using other micron-sized particles, where 5–115 particles/cell were counted^35–38^.

Our results suggest that ScreenMAG particles are good candidates for magnetic hyperthermia against cancer. In vitro*,* magnetic hyperthermia using ScreenMAG particles was remarkably efficient in inducing cell death after 1 h. Moreover, intracellular ScreenMAG are more cytotoxic than extracellular ScreenMAG. This result is in agreement with a previous study^39^. Since the temperature elevation ranged between 43 and 50 °C for intracellular ScreenMAG, the cell death is probably apoptosis as described in Cortie et al.^40^.

This cytotoxic efficiency can be explained by a higher SAR value when the particles are internalized within cells than when they are in suspension. In fact, it has been demonstrated that the SAR value drastically depends on the characteristics of the particles, i.e. size, shape, material, agglomeration state and even on the properties of the dispersion medium^32^. Consequently, two phenomena can explain this increase in SAR of ScreenMAG particles when internalized into cells. The SAR values are dependent on temperature as shown by Garaio et al.^41^ The authors conclude that the SAR values measured at typical hyperthermia temperatures (from 41 to 46 °C) have to be considered, rather than the SAR values measured at room temperature. Here, the SAR of the particles alone was measured from 25 °C, whereas the SAR of the iron-labeled cells was measured from 37 °C. Another explanation is supported by Iacovita et al.^42^. Particles with good dispersion rather than aggregation are beneficial to maintaining a high heating efficiency when they are internalized inside cells. This suggests that the ScreenMAG particles are well dispersed within the cell endosomes, which would increase their heating performances.

The lengthening of T2 after AMF may result from the release of the particles in the extracellular medium by dead cells. Consequently, the local concentration and aggregation state of particles decreased. This effect has already been shown with MPIO^43^ and other IOP. Also, as seen on the MR images in vivo, the IOP continued to be trapped inside the tumors after the MFH treatment. The retention of the IOP within the tumors could be beneficial if numerous AMF applications have to be performed in order to enhance the treatment efficiency^44^. After one MFH application, the ScreenMAG particles were not degraded and no modification of structures or shapes was observed (Supplementary Data S2D), in accordance with previous reports^1^. The application of this repetitive treatment strategy is also supported by histology that showed that the IOP are within cells 7 days after AMF application. Tumor cells may have internalized the IOP that have been released by the dead cells after the first AMF application.

Despite the strong cell death measured in vitro, applying AMF in vivo seemed to induce only a slight slowing down of the growth of an orthotopic glioma model at 42 days. The tumor volumes were heterogeneous, leading to a non-significant difference between the two groups. The SAR calculated for ScreenMAG is high compared to MPIO particles, similar to nanoparticles from Chemicell^45,46^, and is twice as high as the one of Ferumoxytol (60 W g^−1^ ± 17.7)^17^. Our data reveals that the Néel relaxation represents the main contribution (62%) to the SAR of ScreenMAG particles. These results obtained under a simulated viscosity of a biological system can thus not explain the low efficiency of MFH treatment in vivo. Nevertheless, there are possible explanations for the differences between the MFH treatment efficiency in vitro and in vivo. First, the mice used in the current study have immunodeficient systems. Previous studies demonstrated that the immune system is critical to mediate cell death in tumors and tumor microenvironments during therapies^47^. Second, previous studies showed that multiple AMF sessions could be more efficient than a single session to treat tumors^44^. Third, several studies indicate that cancer treatment could be more efficient when magnetic hyperthermia is combined with other therapies like radiotherapy^48–50^. Fourth, the temperature within the tumor could not be monitored during the AMF application. Uncontrolled mild hyperthermia without cytotoxicity could therefore have been generated. Nevertheless, mild hyperthermia combined with radiotherapy has been shown to slow tumor growth^51^. Fifth, the amount of IOP per cell can vary, which leads to variability in SAR when applying the AMF in vivo and, consequently, in tumor growth. A way to quantify the amount of iron per cell has been developed^34^, and this could be used to assess the internalization efficiency before implantation into the animals. Also, in order to quantify the amount of IOP in vivo, Magnetic Particle Imaging (MPI) can be employed^52^. MPI could enable the monitoring of the temperature in the tumor in order to check that 43 °C is reached and maintained for at least 30 min.

One of the limitations of our study comes from the MFH parameters (frequency and amplitude) used. The AMF application should be limited to a safety range of frequencies *f* and intensities *H* due to medical and technical restrictions, as established by the Brezovich criterion. In practice, however, a less rigid criterion exists^53^. In our case, to respect this last criterion with an amplitude of 13.4 kA m^−1^, the maximum frequency *f*_*max*_ must be 373 kHz. Yet, at this frequency, the rise in temperature generated by the ScreenMAG particles is low. The corresponding *H × f* value that we used here (6 × 10^9^ Am^−1^ s^−1^) is just a little above the estimated threshold for biological discomfort (5 × 10^9^ Am^−1^ s^−1^). Nevertheless, when we applied this field on the mouse brain, the temperature of the animal was not modified (data not shown). Also, no behavioral alteration was detected over the weeks of follow-up. Another AMF system with less restricted values of frequencies and amplitudes could be used to evaluate if other combinations of these parameters could generate heat without trespassing the Brezovich criterion.

In conclusion, our results show that micron-sized particles have multiple patterns of cell internalization processes depending on their structures. These particles can be employed for MRI cell tracking and multiple treatment strategies, including magnetic fluid hyperthermia against glioma. The larger size of the magnetic particles used here, for example, enables the more effective use of external magnetic fields for magnetic drug targeting^54–56^.

## Methods

### Iron oxide particles

Two micrometric commercial fluorescent IOP were used: MC03F MPIO-Dragon green from Bangs Laboratories and ScreenMAG green from Chemicell. MPIO (stock solution of 10 mg mL^−1^) and ScreenMAG (stock solution of 50 mg mL^−1^) are carboxylic acid functionalized magnetic fluorescent polystyrene or silica microspheres of 0.98 µm or 1 µm mean diameter, respectively.

The total iron content was determined by inductively coupled plasma-atomic emission spectroscopy (ICP-AES) using Jobin Yvon JY70 + instrument (Longjumeau, France). Prior to analysis, the samples (50 µL) were diluted in a mixture of nitric acid (65%; 600 µL) and hydrogen peroxide (30%; 300 µL), then mineralized by microwave digestion (MLS-1200 Mega, Milestone, Analis, Belgium).

### Magnetic characterization

Magnetic measurements were performed on a Microsense EZ-7 Vibrating Sample magnetometer on ScreenMAG. Magnetization values were normalized respectively to the Fe cation content (265 mM) (see Fig. 1D for further explanation).

### Dynamic light scattering (DLS)

DLS measurements on the ScreenMAG particles diluted in pure water were performed with a fiber optic remote head DLS setup Vasco Flex (Cordouan Technologies, Pessac, France) in cell media at Laboratoire de Chimie des Polymères Organiques, Pessac, France. This system enables the measurement of hydrodynamic diameters even in strongly scattering (turbid) samples (see Supplementary Data S3 for further explanation).

### Negative stain transmission electron microscopy

IOP were diluted in DMEM medium for 15 min, rinsed three times with milliQ water and separated from the supernatant on a strong magnet rack in order to eliminate medium. MPIO or ScreenMAG particles (Fe concentration at 46 mM or 212 mM, respectively) were deposited on a carbon-copper grid pretreated by a standard glow discharge procedure (2 mA, 0.3 m Bar, 40 s, Elmo, Cordouan Technologies) for 30 s. Grids were stained for 2 min with 2% aqueous uranyl acetate. Specimens were then observed with a 120 kV CM 120 (FEI) transmission electron microscope at 3000× and 17,000× magnifications. Image acquisition was carried out using a 2000 × 2000 pixel CCD camera (Gatan). The global diameter has been determined in measuring 40 particles. The sizes of 40 magnetic cores were measured from 10 TEM images.

### Cell culture

U87-MG (human glioblastoma) cells were cultured in Dulbecco’s modified Eagle’s medium DMEM (Life Technologies), supplemented with 1% streptomycin/penicillin/fungizone and 10% fetal calf serum (Life technologies). Cells were incubated at 37 °C in a 5% CO_2_ humidified atmosphere.

### Correlative light and electron microscopy (CLEM)

Cells were seeded (3 × 10^4^ cells per glass bottom gridded dish, MatTek Corporation) in 2 mL of the appropriate growth medium and were grown for one day to reach 40% confluence. MPIO (0.3 mM Fe) or ScreenMAG (3.2 mM Fe) were incubated on cells using complete medium or free-serum medium, respectively (see Supplementary Data S5A for more explanation). After 4 h of incubation, cells were washed with PBS and fixed with 4% PFA in PBS for 2 h at room temperature. Cells were observed in fluorescence microscopy using a Leica DMI6000B microscope equipped with a CCD camera. After washes in milliQ water, cells were post-fixed using 1% (v/v) osmium tetroxide in PBS for 1 h. Cells were embedded in Epon for ultramicrotomy. Ultrathin sections were stained with 2% uranyl acetate and lead citrate before examination using a 120 kV CM120 (FEI) transmission electron microscope.

### Iron quantification

The iron assay kit (Abnova), used in accordance with the manufacturer’s instructions, determined the amount of internalized particles over increasing incubation times.

### Cell viability assay and proliferation test

For cell viability, U87-MG were seeded in 96-well plates (4 × 10^4^ cells/well). The following day, cells were incubated with IOP (MPIO at 0.3 mM Fe or ScreenMAG at 3.2 mM Fe) and different concentrations of chemical inhibitors over 4 h. PrestoBlue Cell Viability Assay (Life Technologies) determined cell survival (see Supplementary Data S5C for more explanation).

To assess the influence of labeling on cell proliferation, U87-MG cells were plated in 4-well plate (2 × 10^5^ cells/well) containing complete medium and ScreenMAG (3.2 mM Fe). Every day, a MTT assay was performed according to manufacturer (See Supplementary Data S5B for more explanation).

### Internalization pathway of iron oxide particles

For incubation time-dependent experiments, cells were seeded on 12-well plates (2 × 10^5^ cells/well) and allowed to adhere over 24 h. Cells were incubated with ScreenMAG particles (3.2 mM Fe) for increasing periods (10 min, 1 h, 2 h, 4 h and overnight) in appropriate medium. After trypsinization, cell fluorescence was analyzed on a GuavaEasyCyte Millipore flow cytometer (GuavaEasyCyte 6-2L). Cells were preincubated with 0.25% Trypan blue for 1 min just before analysis to quench the fluorescence of IOP absorbed on the cell surface.

In order to analyze the cell internalization pathway of the IOP, flow cytometry and TEM were used in presence or not of endocytosis inhibitors. Inhibitors were added at the appropriate concentrations (Supplementary Data S5C), 30 min prior to IOP addition (either MPIO at 0.3 mM Fe or ScreenMAG at 3.2 mM Fe, in the corresponding medium). Cells were then incubated for 4 h and prepared for flow cytometry analysis as described before.

For TEM analyzes, cells were seeded on 4-well plates (9 × 10^4^ cells/well). After 24 h, IOP were incubated into appropriate medium during 4 h. Then, cells were washed with PBS and fixed with 1.6% glutaraldehyde at 4 °C over 2 h. Afterwards, samples were prepared as described in section CLEM.

### Evaluation of stress

Cells were seeded on 6-well plates (8 × 10^5^ cells/well). After 24 h, IOP were incubated into appropriate medium for 4 h or overnight. HSP70 protein levels were evaluated by western blot (see Supplementary Data S4 for more explanation).

### Tumor implantation and labeled cells injection

All experimental protocols were approved by the Animal Care and Use Institutional ethics committee of Bordeaux, France (approval n° APAFIS#17024-2018101009334314). All methods were carried out in accordance with relevant guidelines and regulations, in compliance with the ARRIVE guidelines.

Swiss Nu/Nu mice (20–25 g, Charles River, L’Abresle, France) were implanted with ScreenMAG-labeled (N = 27) or unlabeled (N = 4) U87-MG cells by stereotaxic injection into the striatum as previously described^57^. To do so, the cells were incubated or not overnight with 3.2 mM Fe of ScreenMAG, washed 3 times, centrifugated at 1000 rpm for 5 min and resuspended to reach a concentration of 500,000 cells per 2 µL. The mice were sacrificed when their body weight loss exceeded 15% of their weight at the day of implantation.

### Ex vivo detection of iron

Mice submitted or not to AMF exposure were perfused with 4% paraformaldehyde 1 day or 7 days post-treatment. After extraction of the brains, the tissues were frozen and subsequently cut in 12 μm-thick slices using a cryostat. Iron was detected using an iron stain kit (Sigma) in accordance with the manufacturer’s instructions.

### Magnetic fluid hyperthermia experiments

AMF was generated using a DM3 commercial applicator (NanoScale Biomagnetics) at different frequencies (146 kHz, 217 kHz, 344.5 kHz and 473.5 kHz) and at corresponding magnetic field amplitudes (21.90 kA m^−1^, 20.69 kA m^−1^, 16.23 kA m^−1^ and 13.36 kA m^−1^). A fiber optic sensor (Neoptix) was used to measure the temperature profiles of the IOP suspensions or cell pellets.

IOP suspensions (100 µL diluted in water, 57 mM Fe) were placed at the middle of the two-coil applicator. The temperature profiles of the suspensions were recorded at room temperature every 10 s and the 4 available magnetic field frequencies were tested. A pure water solution was also employed as a negative control. From the temperature curves, the SAR was calculated by:

$\text{SAR}= \frac{\text{C solvent}}{\text{Fe}} \times \frac{\Delta \text{T}}{\Delta \text{t}}$ Where C_solvent_ is the specific heat of the solvent (C_H2O_ = 4.185 J K^−1^ g^−1^ and C_glycerol_ = 2.41 J K^−1^ g^−1^) and [Fe] is the iron concentration (mg mL^−1^), ΔT/Δt is the initial slope measured during the first 60 s of heating (see Supplementary Data S2A for more explanation).

For in vitro experiments, cells were seeded at 9 × 10^5^ cells/60 mm dish. Three conditions were done: (i) 24 h after seeding, the cells were trypsinized and resuspended in 50 µL of DMEM (called “unlabeled cells”); (ii) after 24 h, ScreenMAG particles (3.2 mM Fe) were incubated in free-serum medium over 4 h; the cells were then washed to eliminate IOP excess into extracellular medium. The cells were then trypsinized and resuspended in 50 µL of DMEM (called “intracellular ScreenMAG”); (iii) 24 h after seeding, the cells were trypsinized. Just before AMF exposure, the cells were resuspended in 50 µL of ScreenMAG particles at 3.2 mM Fe in DMEM (called “extracellular ScreenMAG”). The temperature of the cell suspensions was maintained at 37 °C using a water bath. AMF was then applied for 1 h. Before and after AMF, cytotoxicity was measured by reporting the ratio Viable over Dead cells counted with trypan blue (2%, Pan Biotech). Before and after AMF application, the 50 µL suspensions of labeled cells were placed into 1% agarose gel tubes for MRI experiments. Data was measured via at least three independent experiments.

For in vivo experiments, the rectal temperature of the mice was monitored using the fiber optic sensor OTG-M420 (Opsens). AMF exposure was carried out 24 h after tumor cell implantation, over 1 h (at 473.5 kHz; 13 kA m^−1^). Only a subset of the implanted mice (group II) was exposed to AMF (N = 13). Animals of group I (N = 14) served as tumor growth control.

### MRI experiments

The relaxivities (r_1_ and r_2_) are defined as the slope of the linear regression generated from a plot of the measured relaxation rate (1/T_1_ or 1/T_2_) as a function of iron concentration present in the IOP.

Longitudinal relaxation times (T_1_) values were measured at 4.7 T with an Inversion-Recovery scheme followed by a RARE imaging sequence^58^.

Transversal relaxation times (T_2_) values before and after MFH treatment of ScreenMAG-labelled cells were measured at 4.7 T using a Multi-Slice Multi-Echo sequence (TR/TE = 5 s/2.5 ms; 256 echoes; FOV = 40 × 40 mm; matrix = 128 × 64; 1 slice of 5 mm thickness; Acquisition time = 5 min 20 s).

To monitor tumor growth at 7 T, a Multi-Slice 2D T2-weighted sequence was performed on the mouse brain at day 1, 7, 14, 28, 32 post-implantation, using the following parameters: TR/TEeff = 5000/33 ms; RARE factor: 8; FOV = 20 × 25 mm; matrix = 192 × 256; 35 slices of 0.3 mm thickness; Nex = 4; Acquisition time = 10 min 40 s.

### Tumor volume evaluation

Tumor volumes were deduced using Amira Software (Visage Imaging, Germany) by manually drawing Regions Of Interest (ROI) throughout the MR slices containing hypo-intense and/or hyper-intense areas (resulting from IOP or tumor presence, respectively) (see Fig. 5 for more explanation). The volumes of the tumor was performed by a scientist with 10 years’ experience.

The tumor growth inhibition ratio (T/C) was calculated where C and T represent the means of the relative tumor volumes of the control and treated mice, respectively^59^.

### Statistical analysis

For flow cytometry and western blot experiments, data was collected from at least 5 or 3 separate experiments, respectively and expressed as mean ± standard error of the mean. Student t-tests were performed. A P < 0.05 was considered statistically significant.

For in vivo experiments, exclusion criteria was determined based on the T/C as following. For the control group, the animals with T/C below the standard error of the mean T/C calculated at the latest survival time (42 days post-implantation), were excluded, i.e. 2 animals. For AMF group, the animals with T/C above the standard error of the mean T/C calculated at the earliest survival time (28 days post-implantation), were excluded, i.e. 1 animal. A 2-tailed Student’s t-test with Bonferroni correction was used to determine statistical significance between the 2 groups. All statistical analyses were performed with R software (R: A Language and Environment for Statistical Computing, R Core Team, R Foundation for Statistical Computing, Vienna, Austria, 2016, https://www.R-project.org).

## Supplementary Information

Supplementary information.
