# Supplementary material for: Micron-sized iron oxide particles for both MRI cell tracking and magnetic fluid hyperthermia treatment
Source: Sci Rep. 2021 Feb 8;11:3286. doi: 10.1038/s41598-021-82095-6 (PMC7870900; doi:10.1038/s41598-021-82095-6)
Supplement: Supplementary file 1 — Supplementary information. [file 41598_2021_82095_MOESM1_ESM.pdf]

Supplementary data

Micron-sized iron oxide particles for both MRI cell tracking and Magnetic Fluid Hyperthermia treatment

Laurence Dallet[1], Dimitri Stanicki[2], Pierre Voisin[1]
Sylvain Miraux[1], Emeline J Ribot[1*]

[1] Centre de Résonance Magnétique des Systèmes Biologiques, UMR 5536, CNRS/Univ. Bordeaux, Bordeaux, France

[2] Department of General, Organic and Biomedical Chemistry, NMR and Molecular Imaging Laboratory, University of Mons, 19 avenue Maistriau, B- 7000 Mons, Belgium

[*]Corresponding author: Emeline J Ribot
Centre de Résonance Magnétique des Systèmes Biologiques,
146 rue Léo Saignat, 33076 Bordeaux, France
ribot@rmsb.u-bordeaux.fr

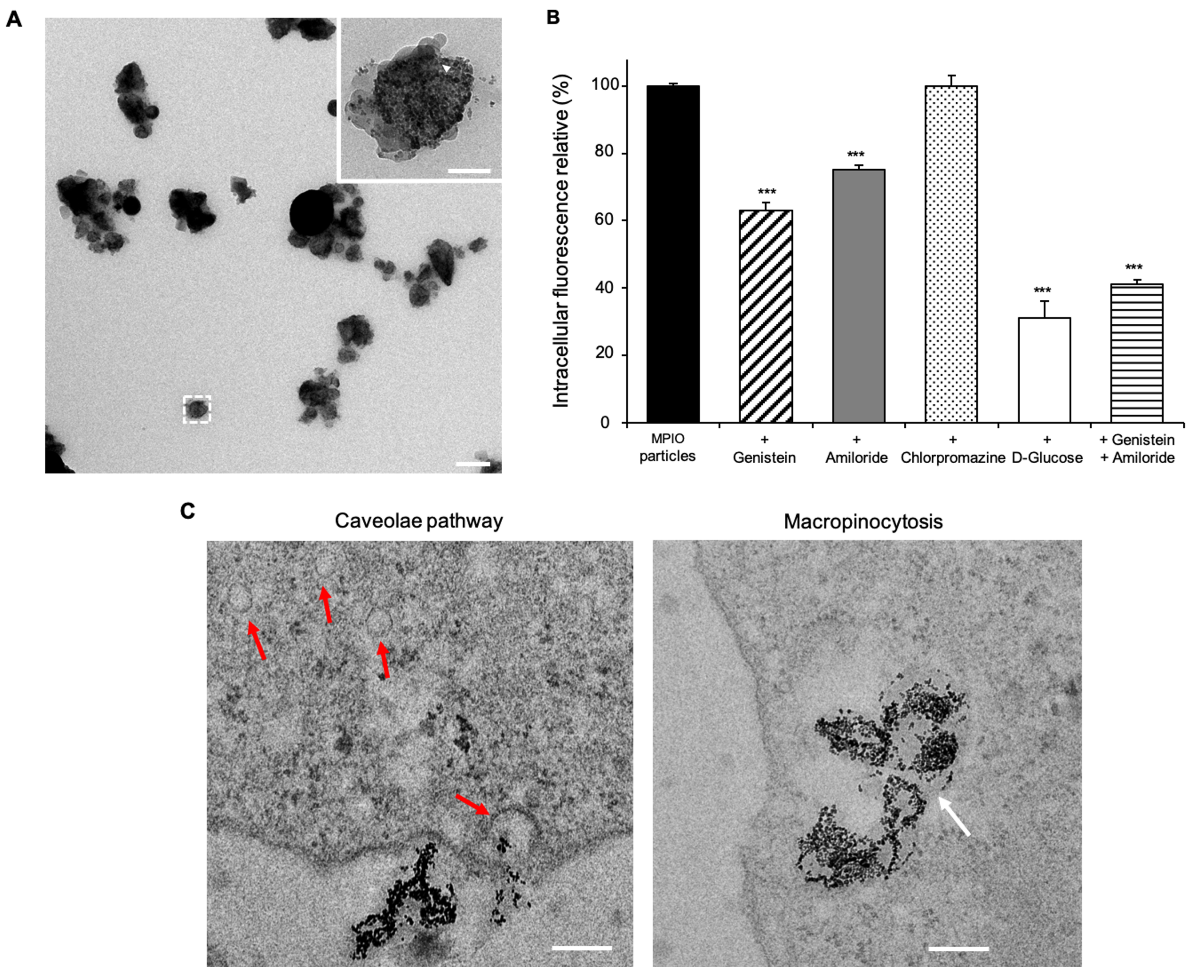

Figure S1: Characterization and internalization pathway of MPIO particles (carboxyl-functionalized magnetic fluorescent polystyrene microsphere; λ_{exc}= 480 nm; λ_{em}= 520 nm).

(A) Transmission electron microscopy images of MPIO. Sizes and shapes were observed in negative staining. Particles are assembled as multi-iron oxide cores inside a matrix. White head arrow shows single iron oxide cores. Scale bar: 500nm (insert: 100nm).

(B) Intracellular fluorescence analysis of iron oxide particles internalization within U87-MG cells treated with various chemical inhibitors (300µM, 10nM, 20µM and 5µM for genistein, amiloride, chlorpromazine and D-glucose, respectively) preventing endocytosis.

*** Significantly different from the control (P≤0.01).

(C) Transmission electron microscopy images of a cell after 4 hours of incubation with MPIO particles. Large vesicles resembling to macropinosomes (white arrow) and very small vesicles resembling to caveolae (red arrows) are observed, enhancing the involvement of macropinocytosis and caveolae pathway to internalize MPIO particles. Scale bar: 200nm.

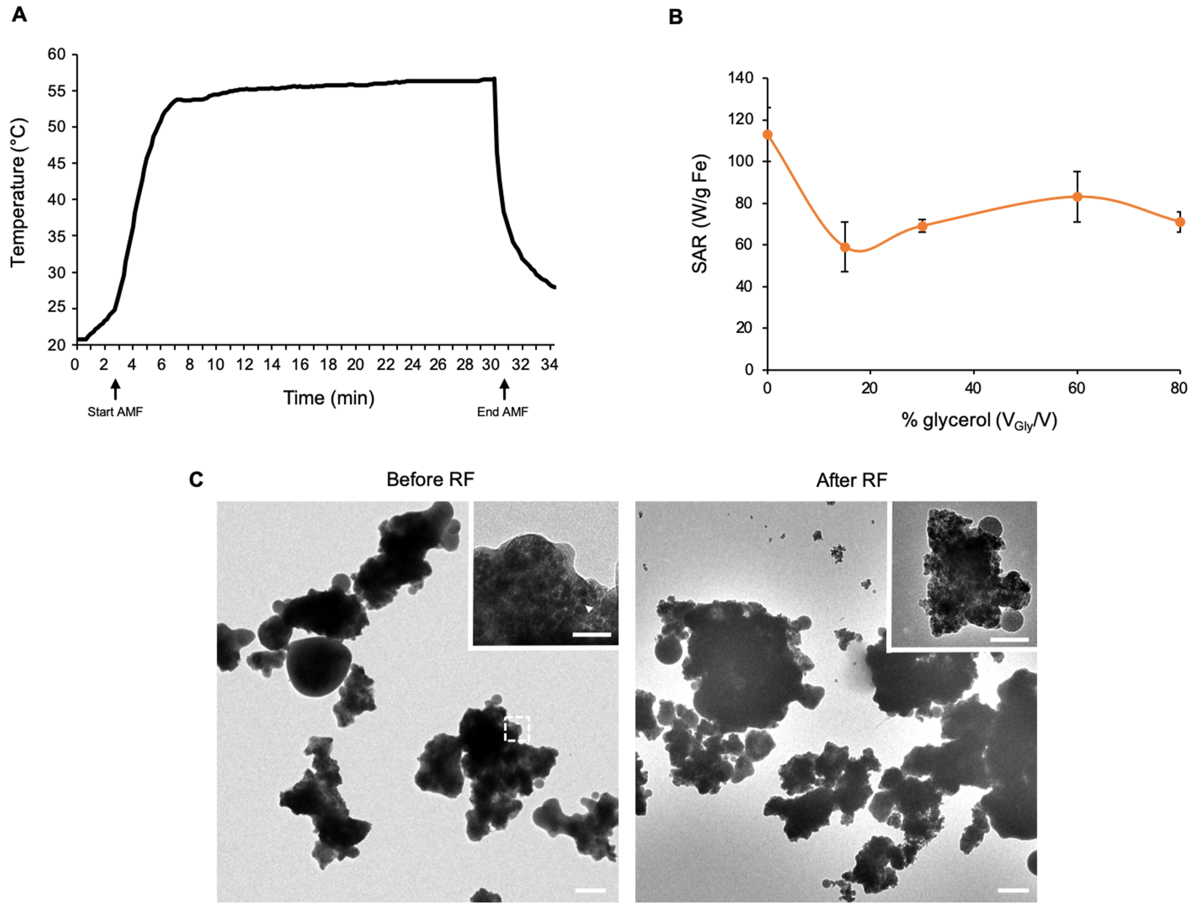

Figure S2: Heating characterization of ScreenMAG

(A) Temperature elevation curve of aqueous suspensions of ScreenMAG at 57mM Fe after application of an alternating magnetic field (AMF) at 473.5 kHz frequency and 13.36 kA/m amplitude. From the temperature curves, the Specific Absorption Rate (SAR) was determined. The SAR is an indicator of the efficiency of magnetic hyperthermia therapy. The temperature of the samples (ΔT) were recorded as function of the AMF application time (Δt). The obtained heating curves were fitted with a linear equation. The initial temperature slope ($\Delta T/\Delta t$) represents the adiabatic case where all the energy is absorbed by the sample and no heat dissipation to surrounding takes place.

(B) SAR values of ScreenMAG dispersed in glycerol solutions at designated viscosity, measured under AMF of 13.36kA/m and 473.5 kHz. Data represent mean ± standard deviation; n = 3.

(C) Transmission electron microscopy images of ScreenMAG before and after AMF exposure. Scale bar: 500nm (insert: 100nm).

A

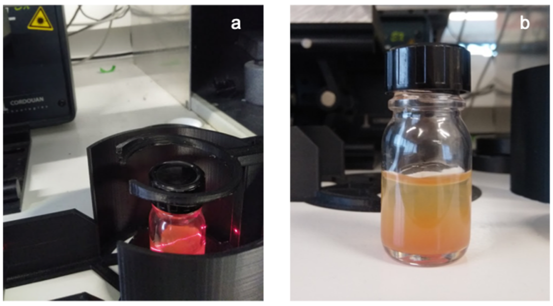

B

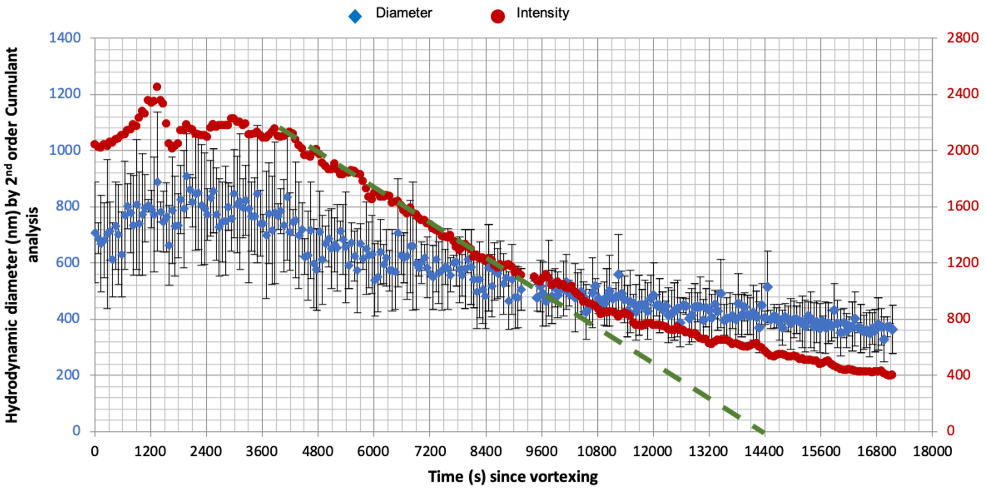

Figure S3: Dynamic Light Scattering measurement of ScreenMAG

(A-a) Photo of sample being measured by DLS. The beam of a laser photodiode at 657nm wavelength (60mW at 100% power) is shined through the sample with a mono-mode optical fiber, and the backscattered light at 165° angle is collected with a second fiber and detected by an avalanche photodiode. Using a second alignment laser, the measured scattering volume was adjusted near the wall of the glass vial (in order to minimize possible multiple scattering effect).The autocorrelation function of light fluctuations $g_2(t)$ is built with a 1000-channel auto-correlator with an adjustable delay time tau. After proper background subtraction from and square root of $g_2(t)$, the autocorrelation function $g_1(t)$ of light amplitude is deduced and analyzed using the standard 2nd order Cumulant fitting method (Johnson, C.S and Gabriel D.A 1995 87-88) leading to the Z-average hydrodynamic diameter using Stokes-Einstein relationship (with solvent parameters taken as those of pure water) and the polydispersity index (PDI). Before measuring the sample, a pure DMEM medium was analyzed but showed not significant background signal. Then 50µL of ScreenMAG-carboxyl beads suspension were diluted in 5mL of DMEM and homogenized by vortexing. A first DLS measurement was done immediately, with 5 sub-runs of 40 sec each, a laser power adjusted at 54% and a tau value of 14µs. Then, measurements were launched continuously with acquisition times of 1 min each for a long period (4h 45 min in the end) in order to follow the evolution of the sample.

(A-b) 5 mL glass vial sample after 5 hours at rest showing partial sedimentation.

(B) Results of kinetic back-scattering DLS analysis of ScreenMAG-carboxy beads diluted 100× in pure DMEM medium: blue diamond markers plot the Z-average hydrodynamic diameter (Z-ave), while error bars have a total width equal to the broadness of the distribution calculated by Z-ave×$PDI^{0.5}$, where PDI (red circles) is the polydispersity index obtained by 2nd order-fitting of the autocorrelograms.

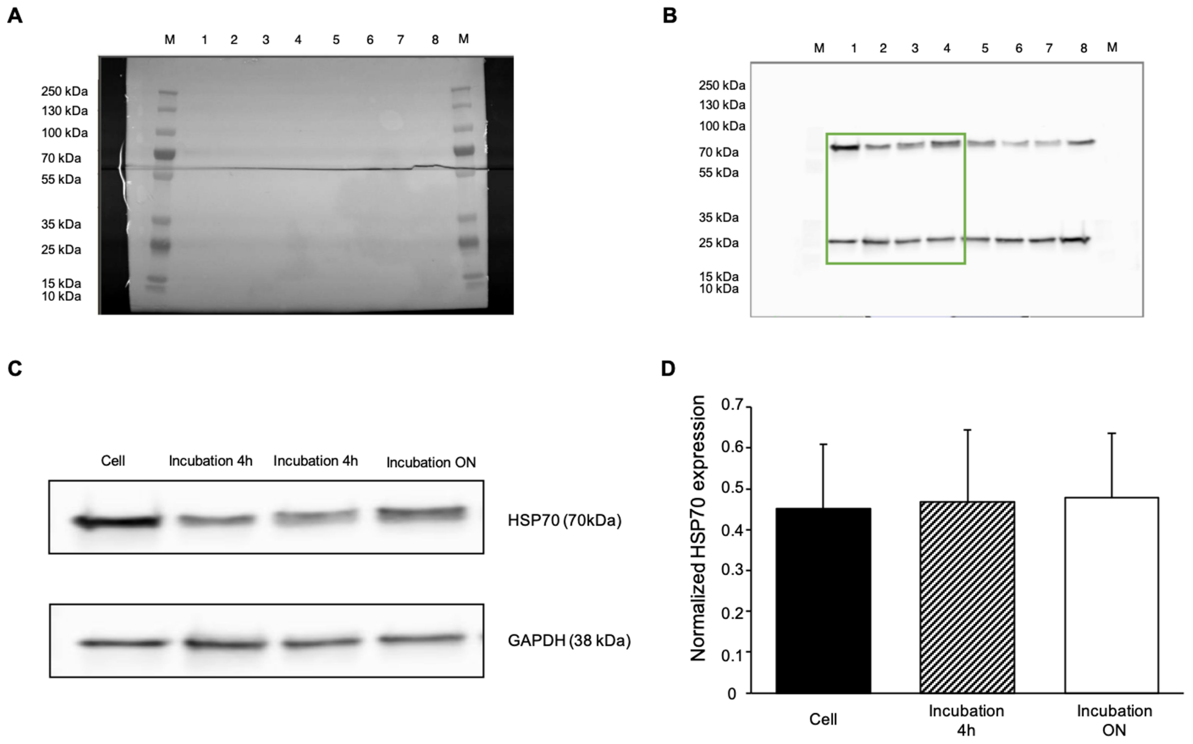

Figure S4: Evaluation of cellular stress by Western Blot.

U87-MG cells were incubated with ScreenMAG particles during 4h or overnight. Pellets were collected and lysed in RIPA buffer according to the manufacturer's instructions (Thermo Scientific). The protein concentrations of each sample were determined by the DC Protein Assay Kit (BioRad). Proteins (40µg) with Laemmli sample loading buffer were resolved on 10% SDS-PAGE (Biorad) and electroblotted onto a nitrocellulose membrane (BioRad). The membrane was blocked for 1h at room temperature with 5% (w/v) skimmed milk in Tris-buffered saline plus 0.1% Tween 20 (TBST). The membranes were then incubated overnight at 4°C in TBST plus 1% skimmed milk containing either anti- HSP70 (Clone C92F3A-5, Invitrogen) diluted at 1:1000 or anti-GAPDH (Santa Cruz) diluted at 1:2000. Next, the membraned were washed three times in TBST, and horseradish peroxidase conjugated secondary antibody anti-mouse (1:4000, Invitrogen) or anti-rabbit (1:5000, Dako) was incubated for 1h. After washing three times in TBST, proteins were detected by incubating the membrane with an enhanced chemiluminescence system (ECL Prime Western blotting detection reagent) according to the manufacturer's instructions, using the Image Quant LAS 4000 mini camera.

(A) Original membrane with weight markers (M) and (B) proteins bands (well1-8). Green square represents proteins bands used for analysis in (C).

1: cell lysate

2 and 3: incubation 4 hours

4 and 5: incubation overnight

6 and 7: incubation overnight +AMF 1 hour

8: incubation overnight + rest 4 hours

(C) Magnification of the green square in (B)

(D) The obtained signals were quantified using the ImageJ software. HSP70 protein levels were normalized to GAPDH expression. These images are representative of 3 independent experiments.

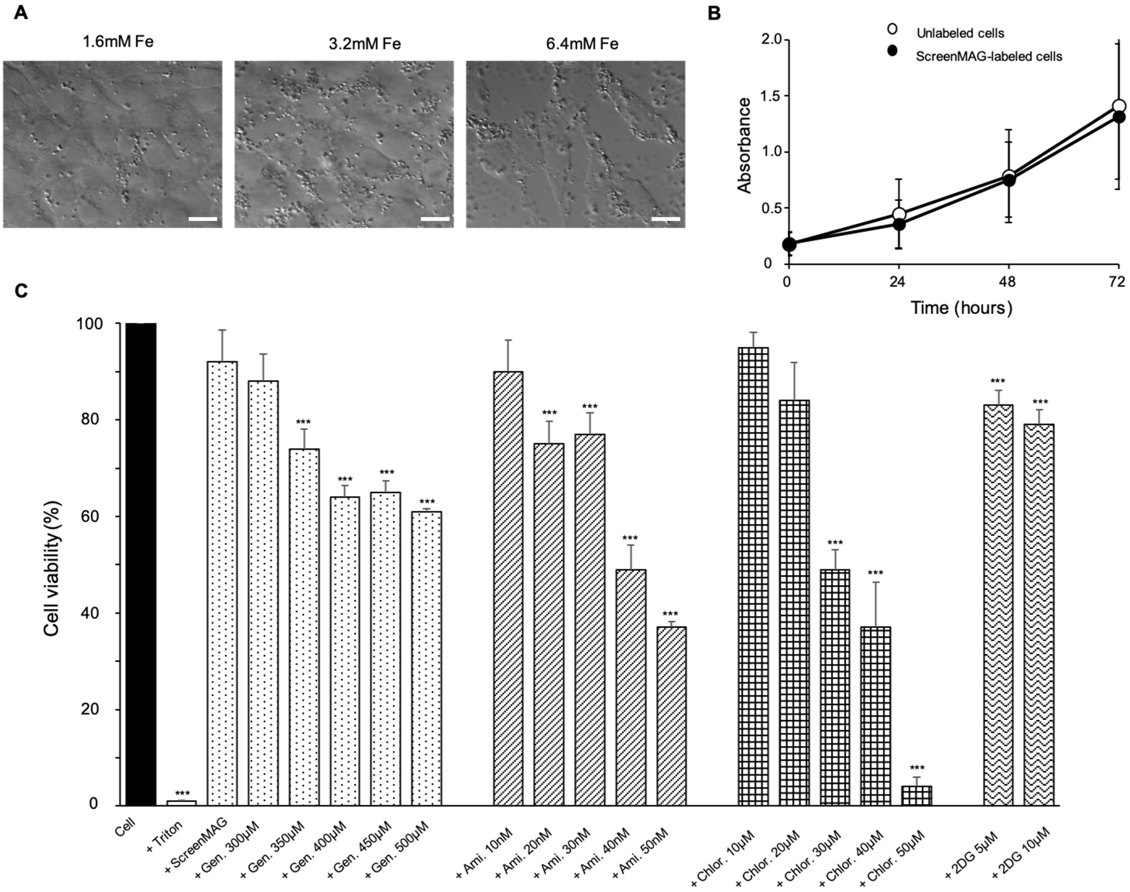

Figure S5: Cell viability and cell proliferation of ScreenMAG particles on U87-MG cells.

(A) DIC images on U87-MG cells after incubation of 1.6, 3.2 and 6.4mM Fe of ScreenMAG particles during 4h. To determine the optimal concentration of ScreenMAG particles to incubate, three concentrations were tested (1.6mM Fe, 3.2mM Fe and 6.4mM Fe). A concentration of 3.2mM was found to generate a high labeling efficiency without cytotoxicity. At lower concentrations, a low amount of particles was internalized. At high concentrations, a decrease of cell density could be observed. ScreenMAG IOPs were incubated into free-serum medium. Indeed, it is well known that the serum alters the internalization of silica-coated particles by creating a protein corona on their surface (Lesniak A. et al. ACS Nano 2012 6:5845-5857). Concentrations and cell culture conditions for MPIO labeling were the same as described previously (Ribot EJ et al. JMRI 2011 34:231-238). Scale bar: 10µm.
(B) Proliferation assay was made on unlabeled and ScreenMAG-labeled cells. 24h after seeding, cells were incubated with ScreenMag at 3.2mM. Then, a MTT assay was performed each day during 4 days like follow. 1mL of MTT solution (0.5mg/mL, Sigma Aldrich) was added per well. After 45min of incubation at 37°C, cells were rinsed with 1mL of PBS and centrifuged (10min). Then, cell pellets were resuspended with 1mL of DMSO. Samples were centrifugated (1000 rpm, 5min) and the optical density at 570nm of the supernatants were analyzed using a spectrophotometer (Hitachi).
(C) U87-MG cells were incubated with a range of concentrations of each chemical inhibitors (300-500µM for genistein, 10-50nM for amiloride, 10-50µM for chlorpromazine and 5-10µM for 2-Deoxy-D-Glucose) during 4h. To determine cell survival, PrestoBlueTM Cell Viability Assay (Life Technologies) was carried out following the manufacturer's protocol. Cells were rinsed with PBS and incubated 2h at 37°C in the reagent diluted in DMEM (1/10). Fluorescence intensity was measured with a fluorescence plate reader (TRIAD LT detector from Dynex). For all conditions, the background control was subtracted before to calculate the ratio (OD sample/ OD control). A concentration of 300µM, 10nM, 20µM and 5µM were determined for genistein, amiloride, chlorpromazine and D-glucose, respectively. Then, a checking that these concentrations allowed to inhibit specifically endocytotic markers of each pathway was performed.
*** Significantly different from the control ($P \leq 0.01$).
